# Associations among resilience, post-traumatic growth, and quality of life in older adults hospitalized with hip fracture: a cross-sectional mediation analysis

**DOI:** 10.3389/fphys.2026.1938367

**Published:** 2026-09-09

**Authors:** Yongning Dong, Jie Peng, Weilin Zhou, Ying Wang, Jiaqing Lu, Yanyun Tan

**Affiliations:** 1 Department of Nursing, Wuxi Huishan District People’s Hospital, Wuxi, China; 2 Department of Emergency Medicine, Wuxi Huishan District People’s Hospital, Wuxi, China; 3 Department of Endocrinology, The Second Medical University Affiliated Hospital of Shandong Province, Weifang, China; 4 Department of Radiology, Affiliated Hospital of Jiangnan University, Wuxi, China

**Keywords:** elderly patients with hip fracture, post-traumatic growth, quality of life, resilience, structural equation model

## Abstract

**Objective:**

This study described post-traumatic growth (PTG), resilience (RI), and quality of life (QOL) in older adults hospitalized with hip fracture and examined their cross-sectional associations, including whether PTG statistically accounted for part of the association between RI and QOL.

**Methods:**

A cross-sectional convenience sample of 308 adults aged 60–90 years with hip fracture was recruited from an orthopedic ward, and 273 valid questionnaires were analyzed (effective response rate, 88.6%). Participants completed the General Information Questionnaire, the 20-item Chinese Post-traumatic Growth Inventory, the Connor-Davidson Resilience Scale, and the Short-Form 12 Health Survey. Associations were evaluated using Spearman correlation, covariate-adjusted linear regression with heteroskedasticity-consistent HC3 standard errors, and a 5,000-resample bootstrap mediation analysis.

**Results:**

Median (interquartile range) scores were 56 (49–60) for PTG, 71 (64–74) for RI, and 41.34 (37.90–44.22) for QOL. RI was positively correlated with PTG (r_s_=0.511) and QOL (r_s_=0.682), and PTG was positively correlated with QOL (r_s_=0.633; all p<0.001). In the fully adjusted QOL model, PTG (standardized β=0.302, p<0.001) and RI (standardized β=0.622, p<0.001) remained independently associated with QOL (R²=0.760; adjusted R²=0.745). The covariate-adjusted indirect association through PTG was 0.131 (bootstrap 95% CI, 0.086–0.175), representing 25.4% of the total RI–QOL association.

**Conclusion:**

RI, PTG, and QOL were positively associated in this cross-sectional sample of older adults hospitalized with hip fracture. PTG statistically accounted for part of the RI–QOL association; however, the simultaneous measurement of all variables precludes causal or temporal interpretation. Longitudinal studies with detailed clinical covariates are required before intervention effects can be inferred.

## 1 Introduction

Population ageing is increasing the number of older adults exposed to frailty, osteoporosis, and fracture-related disability. In 2019, 703 million people worldwide were aged 65 years or older, and this population is projected to reach 1.5 billion by 2050 (United Nations, Department of Economic and Social Affairs, Population Division, 2019). Ageing is also accompanied by greater cognitive, nutritional, and functional vulnerability (Seesen et al., 2021). Osteoporosis is characterized by reduced bone mass and deterioration of bone microarchitecture, resulting in increased bone fragility and fracture susceptibility (Shao et al., 2021; McArthur et al., 2022). Hip fracture is among the most serious osteoporotic injuries because it is associated with substantial mortality, disability, and health-care utilization (Dimai et al., 2022). Its incidence is increasing in China as the population ages (Zhang et al., 2020).

Most older adults with hip fracture receive operative treatment, followed by early mobilization and rehabilitation intended to reduce complications and restore function (Orosz et al., 2004; Wolf et al., 2021). Surgery can facilitate pain control and functional recovery, yet outcomes remain heterogeneous and many patients do not regain their premorbid independence (Schnell et al., 2010; Chen et al., 2018). The burden of osteoporosis and fracture extends beyond the acute injury and may persist throughout hospitalization, rehabilitation, and community recovery (Shen et al., 2022). Consequently, evaluation of hip-fracture recovery should include psychological adaptation and patient-reported QOL in addition to physical outcomes.

Trauma-related adaptation can include both distress and positive psychological change. Some individuals report changes in interpersonal relationships, personal strength, life priorities, and appreciation of life after a traumatic event (Wei et al., 2017; Whealin et al., 2020). These changes have been described after serious illness and hospital discharge (Bonazza et al., 2022; Zhang et al., 2022). RI is related but distinct and refers to the capacity to adapt to adversity, mobilize coping resources, and maintain or regain psychological functioning (Kuranova et al., 2021). PTG was originally conceptualized as positive change arising from the struggle with major adversity (Tedeschi and Calhoun, 1996). Together, RI and PTG provide a framework for understanding why patients facing comparable physical injuries may report different psychological and QOL outcomes.

Conceptually, higher RI may be associated with greater capacity to process the hip-fracture experience, which may be reflected in PTG; PTG may, in turn, be associated with QOL. Studies in cancer, physical disability, spinal cord injury, HIV, and glioma populations have reported positive associations among RI, PTG, and health-related outcomes (Driver et al., 2016; Garrido-Hernansaiz and Alonso-Tapia, 2017; Kim et al., 2020; Dubuy et al., 2022; Fu et al., 2022). Nevertheless, these relationships may differ in older adults with hip fracture because acute mobility loss, dependence, pain, and rehabilitation demands occur together. A focused analysis in this population is therefore warranted.

**Figure 1:**
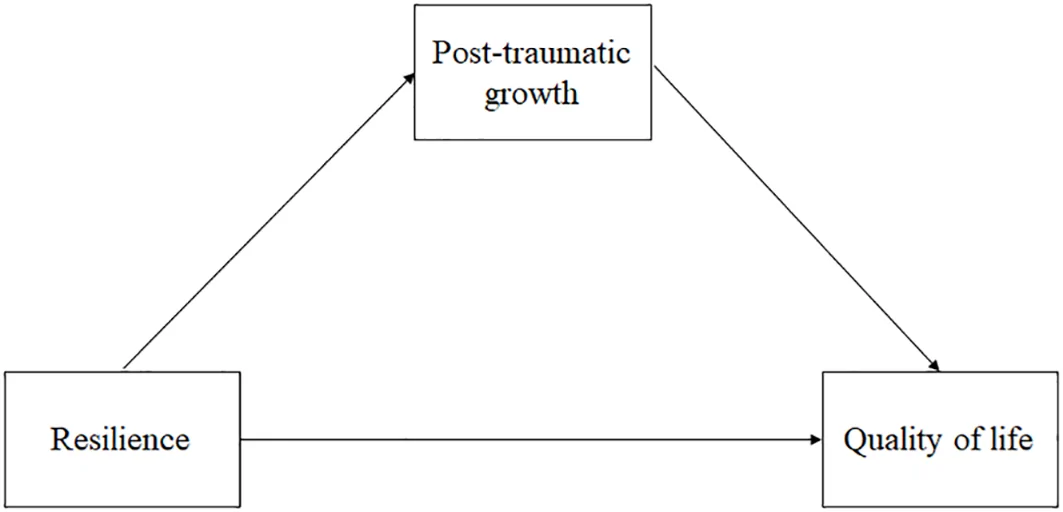
Prespecified cross-sectional mediation model with RI as the independent variable, PTG as the statistical mediator, and QOL as the dependent variable.

Few studies have examined RI, PTG, and QOL together in older adults hospitalized with hip fracture. This study aimed to describe the levels of these variables and evaluate their cross-sectional associations. We hypothesized that (1) RI would be positively associated with QOL and (2) PTG would statistically account for part of the RI–QOL association. **Figure 1** presents the prespecified analytical model. Because RI, PTG, and QOL were measured contemporaneously, the model was interpreted as a cross-sectional decomposition of associations rather than evidence of a causal pathway.

## 2 Materials and methods

### 2.1 Patients

This cross-sectional survey used convenience sampling to recruit patients receiving treatment in the orthopedic ward of a Class III Grade A hospital in Wuxi, China, from January 2022 to March 2023. The target population comprised adults aged 60–90 years who were hospitalized with a clinician-confirmed hip fracture. Inclusion criteria were age 60–90 years, confirmed hip fracture, ability to communicate sufficiently to complete the survey, and provision of informed consent. Exclusion criteria were severe cardiovascular or cerebrovascular disease, impaired consciousness, severe aphasia, or cognitive impairment. Accordingly, the study represents patients who were clinically stable and cognitively and communicatively able to participate rather than the full spectrum of older adults with hip fracture.

### 2.2 Data collection

#### 2.2.1 Research tool

##### 2.2.1.1 The general information questionnaire

The General Information Questionnaire recorded age group, sex, marital status, education, cause of injury, economic source, and economic status. These variables constituted the available covariates for adjusted analyses. Fracture type, operative procedure, comorbidity burden, rehabilitation exposure, and separately measured pain and functional-status variables were not included in the source questionnaire; pain and physical functioning were represented only as components of the SF-12 outcome.

##### 2.2.1.2 Post-traumatic growth

The 20-item simplified Chinese version of the Post-traumatic Growth Inventory (PTGI), adapted by Wang et al. from the original 21-item instrument, was used (Tedeschi and Calhoun, 1996; Wang et al., 2011). The culturally modified version omits the original religious-belief item and contains five domains: personal strength, self-transformation, appreciation of life, relationships with others, and new possibilities. Each item is scored from 0 (no change) to 5 (very great change), yielding a total score of 0–100; higher scores indicate greater PTG. In this study, Cronbach’s α was 0.803 for the total scale. Cronbach’s α values for personal strength, self-transformation, appreciation of life, relationships with others, and new possibilities were 0.783, 0.771, 0.719, 0.788, and 0.770, respectively.

##### 2.2.1.3 Resilience

RI was assessed using the 25-item Chinese version of the Connor-Davidson Resilience Scale (CD-RISC), which comprises tenacity, strength, and optimism dimensions (Connor and Davidson, 2003). Items are scored from 0 (never) to 4 (almost always), producing a total score of 0–100; higher scores indicate greater RI. In this study, Cronbach’s α was 0.839 for the total scale, and the values for tenacity, strength, and optimism were 0.701, 0.787, and 0.872, respectively.

##### 2.2.1.4 Quality of life

QOL was assessed using the Chinese version of the 12-item Short-Form Health Survey (SF-12) (Ware et al., 1996). The instrument covers general health, physical functioning, role limitations due to physical health, bodily pain, role limitations due to emotional problems, mental health, vitality, and social functioning. Standardized domain scores were calculated according to the scoring file used for the study, and the overall QOL score was the mean of the eight domain scores; higher scores indicate better QOL. Cronbach’s α was 0.936 for the scale. The corresponding values for general health, physical functioning, physical role, bodily pain, emotional role, mental health, vitality, and social functioning were 0.931, 0.928, 0.929, 0.934, 0.927, 0.926, 0.934, and 0.934, respectively.

#### 2.2.2 Data collection methods

Trained investigators screened eligible patients, explained the study, obtained written informed consent, and administered the questionnaires. Data were collected once during the index episode of orthopedic care. Face-to-face interviews were used when feasible, and telephone-assisted collection was used when direct completion was not feasible. The General Information Questionnaire, PTGI, CD-RISC, and SF-12 were treated as contemporaneous measures within a single cross-sectional assessment; no temporal ordering among RI, PTG, and QOL was assumed. The source database did not record the exact day relative to admission or surgery, so the assessment cannot be interpreted as occurring at a uniform postoperative time point.

The target sample was estimated as 5–10 participants per observed variable (Zhang et al., 2020). With 23 variables, the initial target range was 115–230 participants; allowing 20% for incomplete questionnaires yielded a planned range of 138–276 valid responses. A total of 308 questionnaires were returned. Thirty-five questionnaires with missing or incomplete responses were excluded, leaving 273 valid questionnaires and an effective response rate of 88.6%.

#### 2.2.3 Statistical approach

Analyses were performed using IBM SPSS Statistics for Windows version 25.0 (IBM Corp., Armonk, NY, USA). Internal consistency was summarized using Cronbach’s α. Because PTGI, CD-RISC, and SF-12 scores were non-normally distributed, continuous variables were summarized as median and interquartile range. Two-group comparisons used the Mann–Whitney U test, and comparisons across more than two groups used the Kruskal–Wallis H test. Associations among PTG, RI, and QOL were evaluated using Spearman rank correlations. For multivariable analysis, QOL was regressed on RI and PTG while simultaneously adjusting for all available prespecified covariates: age group, sex, marital status, education, cause of injury, economic source, and economic status. Categorical variables were dummy coded, with the first category as the reference. Stepwise variable selection was not used. Although ordinary least-squares coefficients were retained for interpretability of the approximately continuous scale scores, inference used heteroskedasticity-consistent HC3 standard errors and 95% confidence intervals. Multicollinearity was assessed using tolerance and the variance inflation factor. Cross-sectional mediation was examined with RI as the independent variable, PTG as the statistical mediator, and QOL as the dependent variable, using the same covariates in all path models. Indirect, direct, and total association estimates were obtained from 5,000 nonparametric bootstrap resamples; an indirect estimate was considered statistically supported when its 95% bootstrap confidence interval excluded zero. All tests were two-sided with p<0.05. The mediation terminology describes statistical decomposition and does not imply temporal or causal effects.

#### 2.2.4 Ethical considerations

The research protocol was approved by the Medical Ethics Committee of Jiangnan University (Approval number: LS2023069). Each patient was informed of the purpose of the study and signed an informed consent form before completing the questionnaire. The privacy of patients is fully respected and protected.

## 3 Results

### 3.1 The general information questionnaire

**Table 1** summarizes participant characteristics. Of the 273 participants, 105 (38.5%) were aged 60–70 years, 77 (28.2%) were aged 70–80 years, and 91 (33.3%) were aged 80–90 years. There were 106 men (38.8%) and 167 women (61.2%); 174 participants (63.7%) had a spouse and 99 (36.3%) did not. Falls accounted for 244 injuries (89.4%) and traffic accidents for 29 (10.6%). Retirement salary was the economic source for 154 participants (56.4%), savings for 70 (25.6%), and family support for 49 (17.9%). Economic status was reported as surplus by 174 participants (63.7%), balanced income and expenditure by 88 (32.2%), and insufficient income by 11 (4.0%).

**Table 1:** General characteristics of participants (N = 273).

Variables	Groups	Number	Percentage, %
Age	60-70	105	38.5
	70-80	77	28.2
	80-90	91	33.3
Gender	Male	106	38.8
	Female	167	61.2
Marital status	A spouse	174	63.7
	No spouse	99	36.3
Education	Illiteracy	19	7.0
	Primary school	80	29.3
	Junior high school	99	36.3
	High school or technical secondary school	48	17.6
	Junior college	20	7.3
	Bachelor degree or above	7	2.6
Causes of injury	Fall down	244	89.4
	Accident	29	10.6
Economic sources	Retirement salary	154	56.4
	Saving	70	25.6
	Family members supply	49	17.9
Economic status	Surplus	174	63.7
	Balance of payments	88	32.2
	Make ends meet	11	4.0

### 3.2 Current analysis of PTGI, RI and QOL

Median (interquartile range) scores were 56 (49–60) for PTGI, 71 (64–74) for CD-RISC, and 41.34 (37.90–44.22) for SF-12 (**Table 2**).

**Table 2:** Descriptive scores for PTGI, CD-RISC, and SF-12 (N = 273).

Variables	Min	Max	Me (IQR)
PTGI	27	71	56 (49–60)
CD-RISC	32	84	71 (64–74)
SF-12	22.06	51.83	41.34 (37.90–44.22)

Statistical method: descriptive statistics; values are median (interquartile range) unless otherwise indicated.Abbreviations: PTGI, Post-traumatic Growth Inventory; CD-RISC, Connor-Davidson Resilience Scale; SF-12, 12-item Short-Form Health Survey; Min, minimum; Max, maximum; Me, median; IQR, interquartile range.

### 3.3 Differences in PTG, RI and QOL on general information

PTG and RI differed by age group and marital status (all p<0.05), whereas QOL differed by age group (p=0.006). The subgroup analyses are presented descriptively in **Tables 3**–**5**; the primary inferential analyses of the associations among PTG, RI, and QOL are reported below.

**Table 3:** Differences in PTG according to participant characteristics (N = 273).

Variables	Groups	PTGI	Statistic	*p*
Age	60–70	56 (51–60)	H=15.862	<0.001
	70–80	58 (53–62)		
	80–90	53 (43.5–58)		
Gender	Male	56 (50–60)	Z=−0.435	0.663
	Female	56 (49–60)		
Marital status	A spouse	56 (51–61)	Z=−2.352	0.019
	No spouse	54 (47–59.5)		
Education	Illiteracy	55 (43.5–59)	H=9.313	0.097
	Primary school	58 (51.75–62)		
	Junior high school	56 (48.5–61)		
	High school or technical secondary school	55.5 (46–58)		
	Junior college	52.5 (49.75–55.25)		
	Bachelor degree or above	55 (52–57)		
Causes of injury	Fall	56 (49–60)	Z=−0.133	0.894
	Traffic accident	56 (52–60)		
Economic sources	Retirement salary	56 (49.25–60)	H=1.186	0.553
	Savings	57.5 (51–61)		
	Family support	56 (42–62)		
Economic status	Surplus	56 (49–60)	H=2.612	0.271
	Balance of payments	57 (50.75–61)		
	Make ends meet	55 (41–58.5)		

Z, Mann-Whitney U test; H, Kruskal-Wallis H Test.

PTG, post-traumatic growth; PTGI, Post-traumatic Growth Inventory.

**Table 4:** Differences in RI according to participant characteristics (N = 273).

Variables	Groups	CD-RISC	Statistic	*p*
Age	60–70	72 (67–74)	H=7.999	0.018
	70–80	71 (66–75)		
	80–90	68 (54.5–73)		
Gender	Male	71 (65–74)	Z=−0.481	0.630
	Female	71 (63.5–74)		
Marital status	A spouse	72 (66.25–75)	Z=−2.514	0.012
	No spouse	69 (61–73)		
Education	Illiteracy	69 (63.5–72)	H=3.653	0.600
	Primary school	71 (62–76)		
	Junior high school	70 (64–74.5)		
	High school or technica secondary school	71.5 (63.75–74)		
	Junior college	72.5 (69–74)		
	Bachelor degree or above	71 (67–72)		
Causes of injury	Fall	70 (64–74)	Z=−0.914	0.361
	Traffic accident	72 (68–74)		
Economic sources	Retirement salary	70 (64.25–74)	H=5.230	0.073
	Savings	72 (67.25–75)		
	Family support	69 (53–74)		
Economic status	Surplus	70 (64–74)	H=4.487	0.106
	Balance of payments	72 (67–75)		
	Make ends meet	68 (61–70.5)		

Z, Mann-Whitney U test; H, Kruskal-Wallis H Test.

RI, resilience; CD-RISC, Connor-Davidson Resilience Scale.

**Table 5:** Differences in QOL according to participant characteristics (N = 273).

Variables	Groups	SF-12	Statistic	*p*
Age	60–70	41.83 (39.25–44.26)	H=10.207	0.006
	70–80	42.30 (38.23–44.89)		
	80–90	40.25 (33.86–42.57)		
Gender	Male	41.36 (39.25–44.41)	Z=−1.173	0.241
	Female	41.29 (37.00–44.17)		
Marital status	A spouse	41.81 (38.25–44.51)	Z=−1.925	0.054
	No spouse	40.96 (37.41–43.09)		
Education	Illiteracy	40.34 (33.10–43.45)	H=6.309	0.277
	Primary school	42.29 (38.03–45.00)		
	Junior high school	41.09 (37.76–44.17)		
	High school or technical secondary school	41.09 (37.19–43.00)		
	Junior college	41.51 (38.43–43.56)		
	Bachelor degree or above	42.22 (41.84–45.49)		
Causes of injury	Fall	41.31 (37.73–44.22)	Z=−0.919	0.358
	Traffic accident	42.55 (39.25–44.39)		
Economic sources	Retirement salary	41.15 (37.63–43.35)	H=3.606	0.165
	Savings	42.20 (39.25–44.38)		
	Family support	42.37 (27.08–45.47)		
Economic status	Surplus	41.28 (37.63–43.61)	H=3.313	0.191
	Balance of payments	42.35 (39.05–44.93)		
	Make ends meet	38.99 (29.16–44.58)		

Z, Mann-Whitney U test; H, Kruskal-Wallis H Test.QOL, quality of life; SF-12, Short-Form 12 Health Survey Questionnaire.

### 3.4 Covariate-adjusted multivariable analysis of QOL

The multivariable model included PTG, RI, age group, sex, marital status, education, cause of injury, economic source, and economic status (**Table 6**). No problematic multicollinearity was detected (maximum VIF = 5.371). PTG (B = 0.227, robust 95% CI 0.155–0.298; standardized β=0.302; p<0.001) and RI (B = 0.383, robust 95% CI 0.323–0.443; standardized β=0.622; p<0.001) were independently and positively associated with QOL. The full model explained 76.0% of the variance in QOL (R²=0.760; adjusted R²=0.745; robust F = 63.638; p<0.001).

**Table 6:** Covariate-adjusted multivariable linear regression of QOL (N = 273).

						95% Confidence interval		Collinearity statistics	
	B	HC3 SE	β	t	*p*	Lower	Upper	Tolerance	VIF
(Constant)	1.378	1.777		0.775	0.438	−2.104	4.860		
PTG	0.227	0.036	0.302	6.227	<0.001	0.155	0.298	0.461	2.169
RI	0.383	0.031	0.622	12.543	<0.001	0.323	0.443	0.459	2.179
Age 70–80 vs 60–70	−1.000	0.610	−0.066	−1.641	0.101	−2.196	0.195	0.747	1.339
Age 80–90 vs 60–70	−0.539	0.568	−0.037	−0.950	0.342	−1.653	0.574	0.670	1.493
Female vs male	−0.951	0.437	−0.068	−2.176	0.030	−1.807	−0.094	0.933	1.072
No spouse vs spouse	0.545	0.509	0.038	1.070	0.285	−0.453	1.543	0.846	1.182
Primary school vs illiteracy	0.901	0.981	0.060	0.919	0.358	−1.022	2.825	0.229	4.362
Junior high school vs illiteracy	1.175	0.994	0.083	1.182	0.237	−0.774	3.124	0.186	5.371
High/technical school vs illiteracy	0.558	1.047	0.031	0.533	0.594	−1.494	2.611	0.263	3.797
Junior college vs illiteracy	0.301	1.508	0.011	0.199	0.842	−2.654	3.255	0.418	2.394
Bachelor or above vs illiteracy	3.516	1.110	0.081	3.169	0.002	1.341	5.691	0.651	1.537
Traffic accident vs fall	0.427	0.510	0.019	0.837	0.402	−0.572	1.426	0.898	1.114
Savings vs retirement salary	1.056	0.630	0.067	1.675	0.094	−0.179	2.291	0.451	2.219
Family support vs retirement salary	1.377	0.927	0.077	1.486	0.137	−0.440	3.193	0.287	3.486
Balance vs surplus	−0.465	0.601	−0.032	−0.774	0.439	−1.643	0.713	0.373	2.680
Make ends meet vs surplus	0.083	1.376	0.002	0.060	0.952	−2.614	2.779	0.607	1.649

QOL was the dependent variable. B is the unstandardized coefficient; HC3 SE is the heteroskedasticity-consistent standard error; β is the standardized coefficient. Reference categories were age 60–70 years, male sex, spouse, illiteracy, fall, retirement salary, and economic surplus. R²=0.760; adjusted R²=0.745; robust F = 63.638; p<0.001.

### 3.5 Correlation analysis of PTG, RI and QOL

Spearman analysis showed positive correlations between RI and PTG (r_s_=0.511), RI and QOL (r_s_=0.682), and PTG and QOL (r_s_=0.633); all correlations were statistically significant (p<0.001; **Table 7**).

**Table 7:** Spearman correlation analysis of PTG, RI, and QOL (N = 273).

	1	2	3
1. CD-RISC total score	1.000		
2. PTGI total score	0.511***	1.000	
3. SF-12 total score	0.682***	0.633***	1.000

all correlations were significant at p<0.001 (two-tailed).

PTG, post-traumatic growth; RI, resilience; QOL, quality of life; PTGI, Post-traumatic Growth Inventory; CD-RISC, Connor-Davidson Resilience Scale; SF-12, 12-item Short-Form Health Survey.

### 3.6 Covariate-adjusted cross-sectional mediation analysis

The prespecified mediation model was evaluated after adjustment for age group, sex, marital status, education, cause of injury, economic source, and economic status. In Model 1, RI was positively associated with QOL (B = 0.514, HC3 SE = 0.020, standardized β=0.835, p<0.001). In Model 2, RI was positively associated with PTG (B = 0.576, HC3 SE = 0.041, standardized β=0.704, p<0.001). In Model 3, both RI (B = 0.383, HC3 SE = 0.031, standardized β=0.622, p<0.001) and PTG (B = 0.227, HC3 SE = 0.036, standardized β=0.302, p<0.001) were positively associated with QOL (**Table 8**).

**Table 8:** Covariate-adjusted regression models for cross-sectional mediation analysis (N = 273).

Model	Model 1		Model 2		Model 3	
Dependent variable	QOL		PTG		QOL	
	B (HC3 SE)	β	B (HC3 SE)	β	B (HC3 SE)	β
RI	0.514 (0.020)***	0.835	0.576 (0.041)***	0.704	0.383 (0.031)***	0.622
PTG					0.227 (0.036)***	0.302
R²	0.718		0.539		0.760	
Adjusted R²	0.701		0.512		0.745	
Robust F	55.037***		22.932***		63.638***	

values are unstandardized B (HC3 robust SE) and standardized β. All models adjusted for age group, sex, marital status, education, cause of injury, economic source, and economic status. ***p<0.001. Stepwise selection was not used.

**Figure 2:**
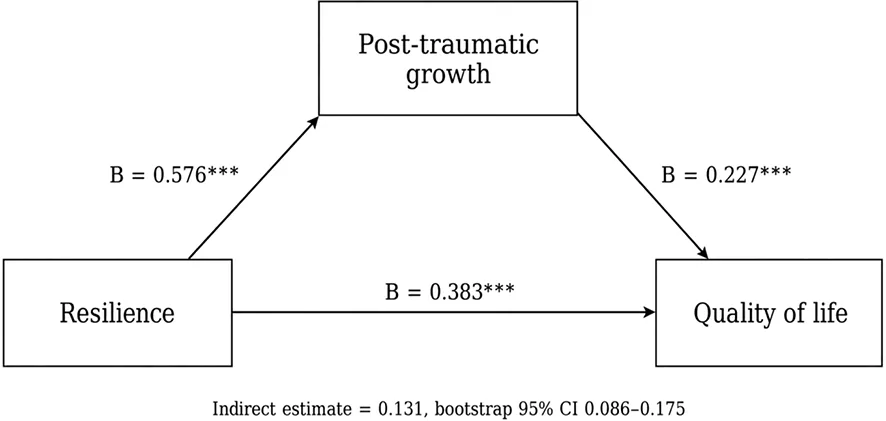
Covariate-adjusted cross-sectional mediation model. Values are unstandardized coefficients estimated with HC3 standard errors; ***p<0.001 (two-tailed); N = 273. Covariates were age group, sex, marital status, education, cause of injury, economic source, and economic status.

The covariate-adjusted indirect association through PTG was 0.131 (bootstrap 95% CI, 0.086–0.175), the direct association was 0.383 (bootstrap 95% CI, 0.324–0.439), and the total association was 0.514 (bootstrap 95% CI, 0.473–0.550). The indirect component represented 25.4% of the total association (**Table 9**). **Figure 2** displays the fitted path coefficients. These estimates support partial statistical mediation in the specified cross-sectional model but do not establish that RI temporally precedes PTG or that either construct causes QOL.

**Table 9:** Bootstrapped direct, indirect, and total association estimates for the RI–PTG–QOL model (N = 273).

Association component	Estimate	LLCI	ULCI	Proportion of total
Indirect through PTG	0.131	0.086	0.175	25.4%
Direct RI–QOL	0.383	0.324	0.439	74.6%
Total RI–QOL	0.514	0.473	0.550	100%

estimates were adjusted for age group, sex, marital status, education, cause of injury, economic source, and economic status and based on 5,000 nonparametric bootstrap resamples.

RI, resilience; PTG, post-traumatic growth; QOL, quality of life; LLCI, lower limit of the 95% confidence interval; ULCI, upper limit of the 95% confidence interval. Cross-sectional statistical mediation does not establish causality.

## 4 Discussion

### 4.1 Current status of RI, PTG, and QOL in older adults with hip fracture

RI can buffer the functional and psychological consequences of adversity (Edwards et al., 2017). The median CD-RISC score in this sample indicated moderate-to-high RI, although the distribution showed substantial heterogeneity. This pattern is consistent with the view that resilience is a dynamic capacity rather than a fixed absence of distress (Nishimi et al., 2021; Guelmami et al., 2022). The median PTGI score indicated a moderate level of reported positive change. PTG may coexist with distress and should not be interpreted as proof that the fracture experience was beneficial (Whealin et al., 2020). QOL was also moderate and remained lower in the oldest age group. Hip fracture can result in prolonged limitations in mobility and independence, recurrent-fracture risk, and sustained treatment and care costs (Magaziner et al., 1990; Magaziner et al., 2000; van Staa et al., 2002; Binder et al., 2004; Moriwaki et al., 2017). These findings support assessment of psychological adaptation and QOL alongside physical recovery.

### 4.2 Cross-sectional associations among RI, PTG, and QOL

RI was positively associated with PTG, consistent with findings in patients with glioma and other trauma-exposed populations (Kim et al., 2020; Lee et al., 2020). In the prespecified model, RI was treated as the antecedent statistical variable and PTG as the mediator; however, the cross-sectional design cannot exclude reciprocal or reverse associations. Higher RI may coincide with more adaptive appraisal and coping, while greater PTG may also reinforce perceived resilience. Positive health-management strategies have also been linked with subjective health and well-being (Yun and Sim, 2021). RI was positively associated with QOL, in line with studies in psychiatric, cardiovascular-fitness, and cancer populations (Hofer et al., 2017; Pozuelo-Carrascosa et al., 2017; Zhang et al., 2017). PTG was independently associated with QOL after adjustment for the available covariates. Prior literature has linked PTG with positive life appraisal and selected physical and mental health outcomes, although the strength and direction of these relationships vary by population and time since trauma (Barskova and Oesterreich, 2009; Bostock et al., 2009; Dougall et al., 2017; Jayawickreme et al., 2021). The bootstrap analysis indicated that PTG statistically accounted for approximately one quarter of the RI–QOL association. This result identifies PTG as a potential clinical assessment target, but it does not demonstrate that increasing RI will cause PTG or QOL to improve. Supportive, cognitive-behavioral, meaning-centered, and pain-focused psychosocial approaches merit prospective evaluation rather than assumption of effectiveness in this population (Park et al., 2019; Warth et al., 2020).

### 4.3 Strengths and limitations

This study jointly evaluated RI, PTG, and QOL in an understudied hip-fracture population and used covariate-adjusted regression with robust standard errors and bootstrap confidence intervals. Several limitations constrain interpretation. First, all variables were measured contemporaneously in a cross-sectional survey, so temporal sequence, mediation causality, and intervention effects cannot be established. Second, the exact timing relative to surgery was not recorded or standardized; PTG, RI, and QOL may vary across hospitalization and rehabilitation. Third, convenience sampling from a single hospital limits generalizability. Fourth, exclusion of patients with impaired consciousness, severe aphasia, cognitive impairment, or communication difficulty means that the sample represents a relatively healthier and more communicatively able subgroup of older adults with hip fracture. Fifth, the source questionnaire did not capture fracture type, surgical procedure, comorbidity burden, rehabilitation status, or independent pain and functional-status measures; residual clinical confounding is therefore likely. Finally, self-reported and telephone-assisted responses may be affected by recall, interviewer, or social-desirability bias. Multicenter longitudinal studies with standardized assessment time points and comprehensive clinical covariates are needed.

## 5 Conclusion

In this cross-sectional sample of older adults hospitalized with hip fracture, RI, PTG, and QOL were positively associated. PTG statistically accounted for part of the association between RI and QOL after adjustment for available demographic, injury, and economic covariates. These findings support integrated assessment of psychological adaptation and QOL, but they do not demonstrate causal pathways or prove that interventions targeting RI or PTG will improve QOL. Prospective studies are required to test temporal relationships and intervention efficacy.

## Data Availability

The original contributions presented in the study are included in the article/supplementary material. Further inquiries can be directed to the corresponding author.
